# Resilience and Intrinsic Capacity in Older Adults: A Review of Recent Literature

**DOI:** 10.3390/jcm14217729

**Published:** 2025-10-30

**Authors:** Gabriela Grigoraș, Adina Carmen Ilie, Ana-Maria Turcu, Sabinne-Marie Albișteanu, Iulia-Daniela Lungu, Ramona Ștefăniu, Anca Iuliana Pîslaru, Ovidiu Gavrilovici, Ioana Dana Alexa

**Affiliations:** 1 Grigore T. Popa University of Medicine and Pharmacy Iasi, 700115 Iași, Romania; 2 Faculty of Psychology and Education Sciences, “Alexandru Ioan Cuza” University, 700506 Iași, Romania

**Keywords:** physical resilience, intrinsic capacity, aging

## Abstract

Aging involves a progressive decline in physiological functions, increasing vulnerability to disorders, functional decline, and disability. Emphasizing resilience and intrinsic capacity offers a proactive framework for promoting successful aging and quality of life. This narrative review selected significant articles published within the last five years on resilience, especially physical resilience, and intrinsic capacity, along with earlier relevant works. Articles were primarily searched in English using PubMed, Google Scholar, and Scopus, employing relevant terms with Boolean operators (“AND”, “OR”). Inclusion criteria included peer-reviewed conceptual, observational, and interventional studies on resilience and/or intrinsic capacity in adults over 60, published between 2020 and 2025, highlighting how the inclusion of geriatric evaluation improves health outcomes. Studies not focused on older adults, outside the date range, or non-English articles were excluded. Out of 145 references, 43 articles met the inclusion criteria. ResEvidence suggests that resilience (a dynamic response to stressors) and intrinsic capacity (baseline reserves across locomotion, vitality, cognition, sensory, and psychological domains) are interconnected, with resilience being associated with better health outcomes, a lower prevalence of chronic diseases, and greater mental health stability. Incorporating assessments of resilience and intrinsic capacity into clinical workflows could support targeted interventions; routine screening may guide personalized exercise and psychosocial plans to help prevent functional decline. Utilizing brief, validated tools (e.g., Short Physical Performance Battery, handgrip strength, Geriatric Depression Scale, brief cognitive tests, and resilience scales) can inform interventions such as physical activity, nutritional support, deprescribing, and psychosocial engagement, which may support healthier aging trajectories.

## 1. Introduction

We live in an aging society, with projections indicating that, by 2030, one in six people will be over 60 years old [1]. This demographic change brings significant challenges for healthcare systems worldwide, not only in managing age-related diseases but also in promoting healthy and successful aging [2]. The World Report on Aging and Health defines aging as “preserving capacity for well-being in older age” [3]. It also represents a period marked by the progressive deterioration of physiological integrity that leads to altered functionality and increased vulnerability to morbidity and mortality [4,5]. On the other hand, the concept of successful aging refers to maintaining mental health and cognitive function, actively participating in society, having strong interpersonal relationships, conserving good overall physical health, and maintaining functional ability—the capacity to meet basic needs, make decisions, and remain mobile [6].

This perspective may overlook the significant role of social determinants such as education, income, ethnicity, access to healthcare, and existing inequities, potentially stigmatizing older individuals with chronic disabilities or illnesses [7].

The classical care model based only on disease management is insufficient to ensure the existence of a generation of functional older adults [8]. Emerging concepts such as resilience and intrinsic capacity (IC) have gained importance in addressing the limitations of traditional models [9].

Resilience in older adults is conceptualized as a multidimensional, dynamic process that enables individuals to withstand, adapt to, and recover from acute and chronic challenges. It encompasses psychological, social, and physiological factors, reflecting not just the ability to bounce back after adversity but also the capacity to evolve and maintain functionality over time [10]. The construct of physical resilience (PR) has surfaced to complete and expand the successful aging model [11,12] and encompasses an individual’s capacity to resist or recover from functional decline following acute stressors, integrating physiological reserve, dynamic recovery trajectories, and whole-person adaptability [13]. In particular, the role of physical resilience [14] and intrinsic capacity has been highlighted as fundamental in understanding not only the aging process but also the capacity for healthy, functional aging beyond the presence of disease.

Integral to age-friendly care is the alignment of services with an older person’s values and care preferences—an approach central to the philosophy of Comprehensive Geriatric Assessment (CGA), which emphasizes collaborative, patient-centered goal settings [15].

Building on the concept of resilience, the notion of intrinsic capacity offers a complementary lens through which to understand healthy aging [16]. Intrinsic capacity refers to a person’s mental and physical abilities, consisting of five interconnected domains: locomotion, vitality, cognition, sensory function, and psychological well-being. This function-based model shifts attention from a disease-centered view of aging, focusing on the positive abilities that sustain engagement and autonomy in later life [17]. Current research indicates that intrinsic capacity serves as a powerful indicator of outcomes such as frailty, functional decline, falls, and mortality.

While resilience portrays the dynamic process of recovery following challenges, intrinsic capacity illustrates the baseline psychological and physiological reserve that facilitates rehabilitation. The dual concepts of resilience and IC promote a more complete and proactive background for supporting function, delaying frailty, and enhancing quality of life in older age (Table 1) [18].

Moreover, the operational definitions of these concepts lack universal agreement, hindering consistent measurement and comparison. While social disparities continue to influence aging trajectories, interventions based on resilience and intrinsic capacity may offer pathways to mitigate these disparities by strengthening individual and community assets. For example, tailored physical activity, nutritional support, and psychosocial interventions could help disadvantaged groups to preserve or enhance their reserves, ultimately promoting healthier and more equitable aging. For instance, community-based multicomponent exercise tailored to socioeconomically disadvantaged populations has demonstrated significant improvements in physical functioning and frailty reduction, effectively narrowing health inequity gaps [9,19]. Similarly, nutritional strategies emphasizing protein supplementation and dietary education have yielded positive outcomes in muscle maintenance and resilience, particularly within low-income groups [20]. Furthermore, culturally adapted psychosocial interventions, including cognitive behavioral therapies and social engagement activities, have been associated with enhanced psychological resilience and reduced social isolation, key social determinants of health disparities [21]. Incorporating these evidence-based pathways underscores the importance of addressing social determinants through targeted resilience and intrinsic capacity interventions, which may ultimately promote health equity and successful aging across diverse socioeconomic contexts.

This review aims to synthesize recent advancements in understanding resilience and intrinsic capacity, emphasizing their interrelationship and potential for guiding targeted interventions. Unlike prior literature that often treats these concepts separately, this work adopts a biopsychosocial framework, demonstrating how integrated assessment and intervention strategies can support functional independence, reduce health disparities, and foster equitable, healthy aging trajectories.

## 2. Methods

**Figure 1:**
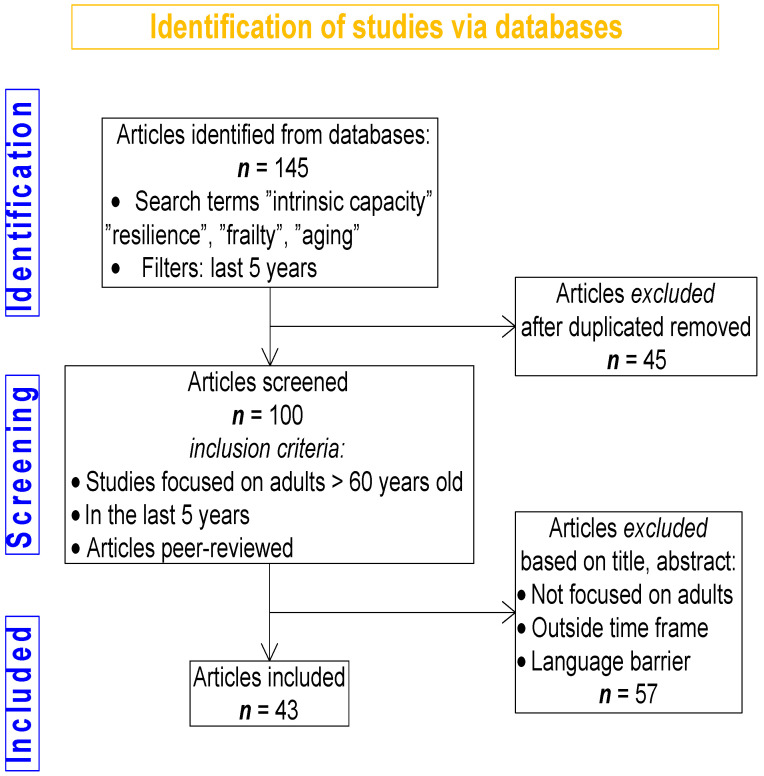
Prisma Flow Chart Diagram.

This research is a narrative review that encompasses a selection of the most significant articles published within the last five years on resilience and intrinsic capacity, alongside earlier significant works considered relevant to the subject matter. Articles were primarily searched in English. Searches were conducted in reputable databases, such as PubMed, Google Scholar, and Scopus, using the following specific search terms: “resilience”, “intrinsic capacity”, “frailty”, and “aging”. To broaden the search, Boolean operators such as “AND” and “OR” were employed to combine search terms and capture a wider range of relevant studies. The inclusion criteria were as follows: peer-reviewed conceptual, observational, and interventional articles, alongside meta-analyses and systematic reviews of randomized controlled trials, addressing resilience and/or intrinsic capacity in adults ≥ 60 years old/older adults from 2020 to 2025. Exclusion criteria included studies not focused on humans and older adults, studies published outside the date range, and studies not available in English. Although 145 relevant references were reviewed and cited for completeness, a total number of 43 articles were included in this narrative review after the application of inclusion and exclusion criteria. A flow diagram illustrating the study selection process is presented in Figure 1.

## 3. Results

### 3.1. Resilience

#### 3.1.1. Definition

Resilience is defined as the ability to effectively adapt to stress, adversity, or trauma, and to recover from challenging experiences while maintaining physical, mental, and emotional equilibrium. It is not a fixed trait but a dynamic process involving a range of cognitive, behavioral, and coping mechanisms that can be learned and developed over time. Windle described resilience as “the capacity for adaptation and bouncing back in the face of adversity” [9]. Resilience is not only the capacity to return to the original state once a stressor is no longer present, but also the capacity to constantly evolve and function efficiently in the face of adversity. Resilience is a key trait that determines the robustness of an organism. This capacity is influenced by intrinsic factors such as optimism and problem-solving skills, as well as extrinsic factors like social support and community resources [22].

According to the National Institute of Aging (NIA), physical resilience—PR—is considered to have real potential in promoting healthy aging [23,24,25].

PR represents the body’s ability to adapt to challenges and recover quickly and completely from illness or other acute stressors like accidents, especially trauma. It expresses the body’s capability to withstand functional decline or to optimize function following a health stressor [26]. As people grow older and progress through life, they may experience a variety of health issues, functional decline, and disability. To address these concerns, healthcare systems must create strategies to promote aging, focusing on psychological, mental, and social well-being in older age [27]. The concept of PR is of great interest for older people facing chronic stressors such as trauma or diseases (e.g., hip fracture, COVID-19) [28].

#### 3.1.2. Understanding Resilience—Theoretical Frameworks

The concept of resilience is not new; it was initially used to describe the elastic properties of materials [10] and consists of multiple elements which combine physical strength with psychological stability. The theoretical foundations of resilience include ecological models and resilience theory, which demonstrate how individuals interact with their environments through dynamic processes. It is a dynamic process with a trajectory marked by positive characteristics throughout life and dynamic responses to stressors.

Adult resilience theory defines resilience as an adaptive process which develops through time, instead of being an innate trait, because it enables people to protect or restore their mental health when facing challenging situations. The development of this process depends on internal assets including optimism, coping mechanisms, and emotional regulation, together with external resources such as supportive relationships and community networks. The Promotive and Protective Factors Model distinguishes between compensatory factors, which directly reduce risk, and protective factors, which act as buffers against risk effects. The COVID-19 pandemic has produced new evidence which demonstrates how personal characteristics interact with environmental pressures and social contexts to determine resilient outcomes [29].

The bioecological theory of Bronfenbrenner serves as the foundation for ecological models which demonstrate that resilience exists within and responds to systemic structures, which range from interpersonal to institutional and cultural levels. Social–ecological perspectives describe resilience as a decentralized complex phenomenon which emerges through cultural interactions between environmental layers. The Resilient Systems Scales measure resilience through life-domain assessments which combine systems theory concepts including engineering stability, ecological adaptability, and proactive responsiveness. These models demonstrate that studying adult resilience needs a systematic approach which considers specific contexts [30].

Later, as adapted in the psychosocial literature, resilience has been defined as the ability to maintain or regain well-being during or after adversity. From the lifespan perspective, resilience is characterized by three essential components: the presence of risk or adversity, positive adaptation or recoil, and protective factors or resources that facilitate successful adaptation in reaction to adversity. Physiological reserve is considered a fundamental determinant of physical resilience [31].

Resilience is a key trait that determines the robustness of an organism to face and overcome adversity amid environmental challenges. As outlined by Karatareos and McEwen (2013), resilience can be explained through the lens of the allostasis model, which describes the body’s capacity to attain stability amidst change [32].

Resilience is imagined as an organism’s capacity to develop adaptive responses—known as allostasis—to environmental challenges and stop these responses once the stressor disappears. This dynamic capacity demonstrates “stability through change”, where flexibility in neural and physiological regulation secures against the cumulative wear-and-tear described as allostatic load.

Individuals exhibiting resilience efficiently use neuroendocrine (e.g., HPA-axis mediators), neurochemical (such as serotonin, neuropeptide Y, and dopaminergic circuits) and neurotrophic factors (including brain-derived neurotrophic factor—BDNF), supporting fear extinction, reward processing, emotional regulation, and social integration. The failure to shut down allostatic responses results in vulnerability which presents as prolonged physiological stress, impaired neural plasticity, and increased risk of psychopathology, including depression and PTSD. Therefore, resilience emerges from efficient allostatic regulation—rapid activation to fulfill a requirement, followed by a quick recovery—a psychobiological indicator distinguishing resilience from vulnerability when facing adversity [33].

This dynamic process allows the brain and body to respond accordingly to internal and external stressors. A person’s biological age increases with chronic low-grade inflammation and individuals with multi-morbidity and high inflammatory markers are susceptible to developing disabilities, needing hospitalization, and dying prematurely [18]. Biological age is directly shaped by resilience-boosting interventions such as proper nutrition, exercise, and vaccinations. An enhanced physical condition provides both improved stress resistance and decreased physiological aging, causing reduced biological age, thus creating a positive feedback loop [34].

#### 3.1.3. Types of Resilience

Based on response time and the nature of the stressor, resilience can be categorized as either acute or chronic. These distinct types, detailed in Table 2, reflect different adaptive responses to varying challenges.

*Acute resilience* is defined as an immediate response to sudden physical stressors, such as recovering from an injury, and chronic resilience which is adapting over time to ongoing stressors like managing chronic health conditions. Acute resilience reflects the capacity for rapid recovery from short-term intense stressors or traumatic situations. It embodies the ability to maintain or rapidly restore physiological and functional homeostasis immediately following an acute adverse event, such as trauma, injury, or acute illness. This resilience is characterized by a dynamic and adaptive process that minimizes the detrimental effects of disruptions and facilitates the swift re-establishment of optimal functioning within a constrained timeframe. In clinical practice, the assessment of acute resilience is indispensable for accurately predicting patient recovery potential and for guiding the development of therapeutic strategies that optimize post-event clinical outcomes. Social support and cognitive flexibility are essential factors that enable rapid psychological stabilization after acute adversity [35].

*Chronic resilience*, in contrast, refers to the sustained capacity of an individual or biological system to preserve functional integrity and adapt effectively in the presence of prolonged, recurrent, or continuous stressors. Unlike acute resilience, which concerns short-term responses to sudden disruptions, chronic resilience reflects long-term adaptability in the face of enduring challenges such as chronic illness, long-term disability, or persistent psychosocial stress. It encompasses the ability to regulate emotional, cognitive, and physiological responses over time to maintain quality of life, psychological stability, and physical independence. This form of resilience is shaped by a complex interplay of internal and external factors, including long-term coping strategies, psychosocial resources (e.g., optimism and self-efficacy), social support networks, lifestyle behaviors, and environmental conditions. Chronic resilience is not a static trait but a dynamic and adaptive process that unfolds across time and context, involving cumulative adjustments in response to evolving stressors. From a clinical perspective, assessing chronic resilience is essential for identifying patients at risk of functional decline and for tailoring interventions aimed at promoting sustained well-being. Various instruments are employed to measure this construct, often focusing on multidimensional aspects such as psychological resilience, life satisfaction, physical endurance, and capacity for long-term self-management [36].

Resilience can be differentiated according to the type of stressor encountered, the adaptative mechanisms involved, and the outcomes observed in cognitive, emotional, behavioral, and physiological functioning. Broadly, resilience is categorized into the following categories: *Psychological resilience* refers to the mental strength required to cope with life’s challenges, including trauma, stress, and significant life changes. Behavioral factors like problem-solving skills, self-efficacy, stress management techniques, and optimism contribute to this type of resilience. Studies frequently examine these factors and utilize scales and questionnaires such as the Resilience Scale (RS) and Connor–Davidson Resilience Scale (CD-RISC) to assess it. Psychological resilience is the most extensively studied type of resilience [37,38].

*Emotional resilience* involves the capacity to recognize, manage, and recover from emotional stressors while maintaining emotional balance despite hardships. Key elements include emotional awareness, mindfulness, regulation, and effective coping strategies. Practices such as mindfulness meditation, reframing thoughts, and processing emotions are studied for their potential to enhance emotional resilience [39,40].

*General health resilience* is a broad concept that includes maintaining or returning to good health across multiple domains (mental, physical, functional) in the face of health challenges. It involves behaviors like physical activity, quality sleep, balanced nutrition, and stress management, as well as social factors such as socioeconomic status, healthcare access, and community support [41].

*Physical resilience (PR)* is the ability to resist functional decline and recover from physical stressors such as illnesses, injuries, or surgeries. It is based on the physiological reserve and the body’s recovery trajectory, with a focus on maintaining independence, especially in older populations. Recovery patterns, cardiovascular health, muscle strength, and overall physical performance are key factors. Interventions like exercise, proper nutrition, and rehabilitation programs are shown to enhance physical resilience [13,42]. Studies articulate PR as a multidimensional, measurable construct linking molecular, system level, and psychosocial domains, which offers potential for focused interventions in geriatric care [43]. PR focuses on older adults’ responses to challenges instead of fixed health markers to understand the intricate relationship between biological processes and environmental factors in aging. This approach offers a more dynamic and equitable method to foster functionality, inclusion, and well-being in late life, by bridging the gaps left by a traditional successful paradigm. Age-friendly care initiatives prioritize environments and services that support older adults’ mental health and autonomy. A study from 2024 proposes a pluralistic framework that integrates the WHO’s Decade of Healthy Ageing priorities—including combatting ageism, fostering age-friendly communities, and integrated and long-term care, anchored in mental health optimization and accessible across community and healthcare domains [44].

*Resilience to Brain Injury*. In the presence of a brain injury, resilience manifests as the capacity to show a better cognitive performance than would be expected. Two types of resilience are identified:

Apparent resilience, where cognitive function stays stable despite specific brain lesions.

Essential resilience, which requires a comprehensive evaluation of the brain conditions and their impact on cognition.

These distinctions help identify individuals with different cognitive decline patterns, influenced by intrinsic factors and social or environmental factors. An understanding of resilience also emphasizes the roles of resistance (the absence of injury) and reserve (pre-injury cognitive capacity) in preventing cognitive decline [42].

Table 3 provides a summary comparison of the resilience types based on stressor, definition, examples, and assessment tools.

#### 3.1.4. Factors Influencing Resilience

Resilience in aging depends on a combination of biological, psychological, and social factors. Engaging in social relationships, employing positive coping strategies, and having a history of adaptive responses to adversity are associated with increased resilience. Studies show that resilient individuals manage stress more effectively and have a lower risk of age-related diseases.

*Biological and Molecular Factors*: Physiological responses such the hypothalamic–pituitary–adrenal (HPA)-axis system influence resilience; aging alters this system, resulting in higher cortisol levels detrimental to cognitive and metabolic health. Maintaining tissue integrity and effective damage control through interventions like exercise and caloric restriction can boost resilience [45].

*Social Factors*: The quality of social relationships and supportive networks significantly enhances resilience. Strong social ties provide emotional resources to help individuals maintain or regain health, mitigating stress effects [13]. *Individual Factors*: Personal traits, such as personality, self-efficacy and coping skills, represent key factors in resilience. High efficacy and adopting a healthy lifestyle (balanced diet and regular exercise) improve resilience [46]. Mental well-being supports effective stress-coping strategies, contributing to overall resilience in aging. Resources, economic stability, and a health-promoting environment (e.g., safe neighborhood with access to healthcare) significantly influence resilience. Supportive environments facilitate resilience development, especially in older populations [13].

Several risk factors negatively influence resilience, such as chronic diseases, especially diabetes and heart disease [8], unhealthy lifestyle choices, lack of exercise, poor diet, psychological stressors, such as high levels of stress, and depression [47].

#### 3.1.5. Multisystem Interactions in Resilience

Biological Mechanisms of Resilience and Their Impact on Health Outcomes: Resilience is a multi-level phenomenon with multisystem interactions integrating neuroendocrine, genetic/epigenetic, neurochemical, and immunological systems. These biologic networks operate in concert to buffer the impact of adversity, with broad implications for both mental and physical health.

*Neuroendocrine Regulation—HPA-Axis Functionality*: The hypothalamic–pituitary–adrenal (HPA) axis serves as the central orchestrator of stress adaptation. Stress triggers corticotropin-releasing hormone (CRH) release from the hypothalamus, driving adrenocorticotropic hormone (ACTH) secretion and subsequent glucocorticoid (e.g., cortisol) release from the adrenal cortex. Dysregulated feedback in the HPA axis is linked to conditions like chronic stress, depression, and increased cardiovascular risk. Resilient individuals exhibit efficient activation followed by swift termination of HPA signaling via intact glucocorticoid receptor (GR) and mineralocorticoid receptor (MR) feedback loops, minimizing prolonged cortisol exposure. Dysregulated feedback, marked by GR resistance or slow termination, is associated with chronic inflammation, hippocampal atrophy, metabolic disruption, and heightened psychiatric and cardiovascular risk. Furthermore, dehydroepiandrosterone (DHEA), exhibiting antiglucocorticoid properties, can offset cortisol’s effects during acute stress, with higher DHEA/cortisol ratios correlating with greater resilience in military and PTSD populations [48].

*Genetic and Epigenetic Modulation—FKBP5, BDNF, and Beyond*: Resilience is partly rooted in genetic and epigenetic modulation of stress-responsive genes; FKBP5, a cochaperone in the GR complex, regulates receptor sensitivity. Certain FKBP5 genotypes promote adaptive GR responsiveness and efficient HPA-axis recovery, while others foster resilience through enhanced HPA flexibility and exploratory phenotypes demonstrated even in avian models. The inhibition or absence of FKBP5 in rodents facilitates hippocampal neurogenesis and improved emotional recovery after stress. Brain-derived neurotrophic factor (BDNF) is a key molecule that may be used for understanding diseases like neurodegenerative disorders. BDNF represents a quintessential factor in neuroplasticity. Reduced BDNF levels are associated with cognitive decline and increased susceptibility to depression, while increased BDNF promotes neuroplasticity and resilience. In hippocampal models, higher BDNF levels foster stress resilience, supporting synaptic remodeling and HPA control, while chronic stress-induced attenuation of hippocampal BDNF undermines these adaptive processes. Conversely, stress-induced elevation of BDNF in the nucleus accumbens promotes depression-like phenotypes, indicating that the spatial regulatory context of BDNF is essential.

Epigenetic modifications including DNA methylation, histone acetylation (e.g., via HDAC interaction), and microRNA regulation (e.g., miR-135, miR-124) play a decisive role in fine-tuning stress-response genes. Early-life experiences can leave lasting epigenetic marks on glucocorticoid receptor or BDNF promoters, influencing lifelong resilience trajectories [49].

*Neurochemical Systems—Serotonin, NPY, Dopamine and Neuropeptides*: Multiple neurotransmitter systems act in synergy to buffer stress responses. Neuropeptide Y (NPY) exerts anxiolytic and cognitive-enhancing effects under stress, counteracting CRH action in limbic regions. Elevated NPY is consistently associated with superior performance and reduced PTSD incidence in high-stress cohorts. The serotonin and dopamine systems regulate coping strategy and reward processing. Genetic variations in SLC6A4 (serotonin transporter), along with the frequency of receptor subtype expression, influence stress reactivity and coping, though mechanistic clarity remains in progress. Dopaminergic resilience pathways controlled by BDNF in the mesolimbic circuit (VTA-NAc) distinguish stress-susceptible from resilient phenotypes in preclinical models [50].

#### 3.1.6. Immunomodulation: Balanced Neuro-Immune Interplay

Several biomarkers of inflammation (TNFR-I, TNFR-II, sVCAM-1, and IL-6), of metabolic and mitochondrial function (non-esterified fatty acids, lactate, ketones, acylcarnitines, free amino acids, and IGF-1), and of epigenetic dysregulation (circulating microRNAs) are discussed as relating to physical resilience in older adults. The association is especially significant following hip fractures. The most cited biomarkers include TNFR-I and specific microRNAs, linked to inflammatory and metabolic signaling channels relevant to biological aging [51].

Resilient phenotypes maintain balanced inflammatory responses. Acute stress can transiently enhance immune activity, but resilience prevents chronic microglial activation, monocyte infiltration, and NLRP3 inflammasome signaling in the brain. Elevated basal pro-inflammatory markers (e.g., IL-1β, IL-6) are typical in individuals vulnerable to depression, while resilience aligns with lower levels and immune-quiescent microglia. Therapeutic immune modulation, for instance, via anti-cytokine treatments, can shift individuals toward more resilient outcomes in stress-related disorders [52].

*Integration and Health Outcomes*: Collectively, these systems shape a phenotype of resilience characterized by rapid HPA-axis reactivity with efficient termination, epigenetically regulated stress-gene profiles supporting neural-based adaptability, neurochemical buffering via NPY, serotonin, dopamine, and neuropeptides, and controlled inflammatory responses, limiting detrimental CNS and systemic effects.

Clinical correlates are compelling: resilient biological signatures predict lower PTSD and depression risk, reduced cardiovascular disease burden, improved recovery from stress exposure, and enhanced longevity with decreased allostatic load [48,53].

Moreover, psychosocial interventions, such as cognitive behavioral therapy and mindfulness, enhance immune function, attenuate the cytokine burden, and strengthen neuroendocrine regulation, with effects lasting months post-intervention [54].

The neurobiological mechanisms supporting resilience are underpinned by complex interrelations of neural and hormonal systems that are responsible for maintaining homeostasis and adaptation, securing functioning and survival. The role of the brain in resilience is of great significance, representing the primary organ that recognizes environmental threats and arranges adaptive responses. Neuroendocrine mechanisms, such as releasing glucocorticoids during stress, indicate how the brain regulates behavior and physiological responses to sustain stability. Early childhood experiences are essential for the evolution of resilience; it is hypothesized that adverse childhood occurrences may interfere with an individual’s capability to adjust to stressors in adulthood. Neuroplasticity is significant as the process that involves adaptive functional and structural changes to the brain; even those who experience early adverse events can still be able to recover and adapt later in life [55].

Hormonal fluctuations can cause alterations in the nervous system, influencing behavior, cognition function, and emotion regulation. While repeated exposure to stress hormones is destructive, the brief activations of these pathways can consolidate resilience by facilitating adaptive coping strategies. The capacity to adapt can adjust through different life stages and is affected by personal experiences and environmental factors, emphasizing the need to distinguish between acute and long-term resilience [56].

Another perspective is the idea of biological embedding, whereby both negative and positive experiences are encoded in the development of neural systems. Exposure to stressors at critical stages results in long-term alterations in brain architecture, affecting an individual’s strength to cope with stressors in the future. Neurobiological models have established that environmental contexts and genetic factors play key roles in this embedding process, resulting in a variety of responses to stress that can predispose individuals to a particular set of health outcomes in adulthood.

#### 3.1.7. Physical Resilience (PR) and Frailty

PR and frailty are interconnected concepts, though they differ in essence. *Frailty* is not simply the opposite of resilience; on the contrary it exists as a distinct condition from resilience because it represents the vulnerability to stressors which develops from aging-related decline that leads to higher chances of negative outcomes. Frailty appears mostly during the later stages of life, whereas resilience operates as a continuous spectrum that functions across the entire lifespan. Due to the varying rates of molecular decline, some individuals may be more prone to stress that others. The shift from a robust state to a fragile state is influenced by an individual’s physiological capacity when responding to stressors, with PR reflecting the actualization of this capacity [57,58].

In comparison with frailty, the resilience framework presents more beneficial intervention possibilities because it provides an encouraging outlook on aging processes and adaptive mechanisms. It focuses on four aspects: resistance (the ability to retrieve normal function after a stressor), latitude (the system’s capacity to sustain deviations from the normal), panarchy (describes system interconnections), and precariousness (measures the distance to an irreparable change point of the system). These characteristics can be assessed by quantifying the magnitude of the stressor, the functional change extent, the recovery speed, and the impact on the system [29]. Studies that used the 15-item modified Activities of Daily Living (ADL) index for assessing resilience in response to acute illness prior to hospitalization showed that recovery typically begins during hospitalization, with frail individuals being particularly vulnerable to such declines. It emphasizes the significance of identifying frailty upon hospital admission to evaluate physical resilience. There are studies that outline that measuring both frailty and dynamic factors, such as pain and step count, can enhance predictions of recovery following acute illness. The findings suggest that improved frailty identification can inform therapeutic decision-making, discharge planning, and rehabilitation strategies [59].

#### 3.1.8. Measurement of Resilience

Resilience encompasses the combined vital physiological reserves that support recovery, essential for promoting independent living and healthy aging. Some studies explore how dynamical and biological system models can quantify and predict physical resilience. Considering the body’s adaptability to changes, these models offer a quantitative approach to assessing resilience. Through the application of dynamical systems theory, researchers can obtain insight into the underlying mechanisms of resilience, which can lead to tailored interventions [60].

A recent study on resilience in Chinese older adults proposed the Clinical Physical Resilience Assessment Scale (CHEES) to measure PR in response to health-related stressors effectively. The researchers identified key items that reflect the capacity of older individuals to recover from challenges, emphasizing that 14 specific items categorize PR into IC, adaptation to change, and external support. The resulting scale demonstrates good reliability and correlation with physical function, addressing a significant gap in assessing resilience as it applies to the aging population [61].

PR has been proposed to be a key factor in deciding whether an older person can achieve successful aging. Therefore, it is essential to develop instruments for assessing the PR of an individual objectively. To operationalize the PR construct, the elements of the triad—system, stressor, and status—should be specified, and the measurement should be simple. Understanding the mechanisms by which a person can physically recover from an acute illness or injury is valuable for identifying people at increased risk of low resilience [62].

To facilitate better understanding, Table 4 summarizes various assessment tools and methods used to evaluate intrinsic capacity and physical resilience.

The measurement of resilience in older adults typically combines self-report questionnaires: gathering self-reported health data using tools like the SF-36 survey [63,64] or the ConnorDavidson Resilience Scale [65] and biological markers such as hormonal responses and epigenetic markers (e.g., cortisol levels and inflammatory cytokines) [66,67]. The assessment of physical resilience includes functional assessments together with performance-based tests which measure gait speed and grip strength [57]. Neuroimaging techniques including MRI have been used to study neural indicators of resilience [68].

Colón-Emeric et al. examined methods to measure PR in older adults after acute stressors using the ‘recovery phenotype’ and ‘expected recovery differential’ approaches. The recovery phenotype method groups patients by recovery patterns and outcomes across multiple measures, identifying younger, healthier individuals as resilient. In contrast, the expected recovery differential focuses on patients recovering better than predicted based on their clinical characteristics, often highlighting resilience in older individuals with more comorbidities. The application on viral respiratory infection and hip fracture cohorts showed moderate agreement between the two methods, suggesting different utilities: the recovery phenotype for clinical prognosis and the expected recovery differential for uncovering biological mechanisms [69].

Currently, the most-used method of measuring PR is monitoring the trajectory toward recovery after stressors. Whitson et al. suggest three possible ways of measuring PR: direct observation of the functional trajectory after the action of a stressor; a trajectory that does not change represents the resilience indicator; and identifying aging phenotypes such as frailty or fatigue to assess PR. ‘Fragile versus robust phenotypes’ is considered the expression of underlying levels of low versus high physical resilience; the difference between chronological age and biological age is assessed by physical tests or biomarkers; younger biological age than chronological age indicates preservation of reserves, indicating resilience in the face of aging.

The quantification of PR continues to be an abstract task, remaining an active research domain [13]. Another method used to assess PR is the Physical Resilience Scale, aimed at measuring PR in older adults, particularly in relation to recovery from hip fractures. Resnick et al. tested on a sample of 130 older adults a repeated-measures design and various resilience assessments (Physical Resilience Scale, Hardy–Gill Resilience Scale, 14-item Resilience Scale, 5-item Geriatric Depression Scale, and the Yale Physical Activity Survey) to provide the scale’s reliability and validity. Findings registered strong psychometric properties, suggesting that the Physical Resilience Scale effectively captures the ability of older adults to withstand and recover from physical health challenges, thus facilitating interventions that could promote successful aging [70].

The Physical Resilience Scale and the CHEES (Cognitive Health and Emotional Engagement Scale) exhibit strong psychometric properties, indicating their reliability and validity in assessing resilience. While the CHEES has been validated within Chinese populations, its cultural specificity necessitates cautious generalization and a context-specific selection of assessment tools [71].

Culturally adapted tools, grounded in local norms, yield more accurate assessments of resilience and intrinsic capacity in older adults.

### 3.2. Intrinsic Capacity—Conceptual Framework

Intrinsic capacity consists of multiple interconnected dynamic elements that combine cognitive abilities with psychological aspects, physical attributes, and social competencies. Functional capacity results from the interaction between intrinsic capacity and the environment. The World Health Organization (WHO) defines it as a critical factor to determine health status and functioning, especially in older people. According to the Integrated Care for Older People (ICOPE) guidelines developed by the WHO, intrinsic capacity (IC) represents the amalgam of physical and mental abilities represented by five important domains: locomotion, cognition, vitality, psychology, and sensory. Like fragility, where there is only one state of fragility at a given moment, intrinsic capacity presents a single global value at a specific time [72,73].

The decline in IC, referring to cognitive impairment, depressive symptoms, or sensory loss, diminishes this reserve and increases vulnerability to external stressors [74].

The development of IC is shaped by biology and psychological and social factors, which makes it consistent with a biopsychosocial model of aging. The relationship between IC and environmental supports demonstrates that healthy aging depends both on individual capability and accessible enabling environments. Recent geriatric paradigms emphasize intrinsic capacity (IC) and resilience, moving beyond deficit-focused models like frailty, positioning IC and resilience as holistic, dynamic elements capturing the heterogeneity of aging at individual levels. Expounding on this, Chhetri et al. conceptualize IC as an integrative proxy for physiological reserve and a foundational determinant of physical resilience (PR), the ability to withstand and rebound from stressors [62].

#### 3.2.1. Factors Influencing IC—Biopsychosocial Mechanisms of IC

Intrinsic capacity (IC) represents a comprehensive measure of an individual’s mental and physical capacities, and it is principally influenced by biological mechanisms like cellular factors, genetic factors, and physiological functioning, in general. Studies demonstrate the heritability of different IC elements, with estimates varying from 20% for sensory capabilities to 85% for locomotor function, indicating a notable genetic influence. Furthermore, the assessment of specific genetic factors remains insufficient, with only a few gene studies on the connection between IC and ApoE gene variants beginning to reveal the genetic grounds [75].

Individuals with low IC presented hyperglycemia, high levels of inflammatory markers such as interlekin-6 (IL-6), plasma C-reactive protein (CRP), monocyte chemoattractant protein-1 (MCP-1), tumor necrosis factor receptor-1 (TNFR-1), growth differentiation factor-15 (GDF-15), and low serum levels of dehydroepiandrosterone sulfate (DHEA-S) [76].

Other factors that greatly influence IC are represented by psychological factors such as cognitive functions, mental resilience, and emotional well-being [77]. Cognitive performance, which includes memory, attention, and executive functions, determines a person’s capacity to adapt and respond to daily necessities and it is indispensable for preserving IC. Psychological resilience, described as the ability to recover from stressors, plays a decisive role in shaping intrinsic capacity [78]. Studies show that intrinsic capacity is a predictive factor of psychological outcomes, counting general life satisfaction levels and mental health disorders [79].

Social factors strongly impact IC, emphasizing the essential relations between a person’s intrinsic abilities and their environment. The quality of social interactions, social support systems, and community engagement plays an essential role in sustaining and enhancing IC. Socioeconomic factors, such as income, education, and access to healthcare, further exert a dual impact on IC by either providing the necessary resources for a heathy life or, conversely, inducing its decline when inadequate. A socially engaging environment protects against isolation-related health problems, while inclusive societal systems enable older adults to keep their functional independence at its best [80].

Research on gene–environment interactions, lifestyle factors, and psychological and social factors hold the potential of improving healthcare interventions to support successful aging, through understanding how IC can be enhanced [76].

#### 3.2.2. Measurement of Intrinsic Capacity

The measurement of intrinsic capacity presents a significant challenge to researchers. The WHO tackles this challenge by creating standardized measurement tools which healthcare professionals can use to evaluate IC with accuracy. The proposed evaluation instruments focus on measuring the five intrinsic capacity domains, which include cognition, psychological function, locomotor ability, vitality, and sensory functions. The assessment tools enable healthcare providers to track intrinsic capacity changes throughout time while enabling prompt intervention strategies. Intrinsic capacity has been proposed as a multifaceted indicator of health. It is implemented to assess physical and mental capacities that are vital for older people to continue performing their most essential activities that they value most. The assessment of IC requires evaluating each of the five domains (cognitive, psychological, sensory, locomotor, and vitality) [81], as follows:

1. *Cognitive*: Cognitive evaluation consists of a test battery that includes four components: (a) temporal orientation; (b) delayed recall of 10 common words (episodic memory); (c) a semantic memory questionnaire (based on four questions about general knowledge); and (d) a semantic verbal fluency task (executive functioning, vocabulary size, and lexical access speed). There are authors that use the MMSE test as a quantification of cognitive status [82].
2. *Psychological*: The GDS stands as the most frequent tool used for evaluating depressive symptoms in patients. There are also scales to evaluate sleep quality, a key contributor to mental health outcomes [83].
3. *Sensory*: To evaluate hearing/visual acuity, individuals are asked basic questions to rate their self-assessment of hearing and vision abilities, e.g., “How do you rate your hearing capacity (even when using a hearing device)?” and “How good is your eyesight?”. Participants responded to these questions by selecting from five options: “excellent”, “good”,” fair”, “poor”, or “very poor” [84].
4. *Locomotor*: Balance and gait speed are usually used to estimate lower limb physical functionality. Balance evaluation uses the SPPB (Short Physical Performance Battery), while gait speed is calculated by measuring the time to walk three meters at the usual pace either with or without assistance devices.
5. *Vitality*: The vitality domain combines various underlying factors including nutrition, alongside strength and energy levels. The nutritional status is determined with the help of an MNA test, the handgrip strength of the dominant hand is measured using a dynamometer, and the level of energy is evaluated by asking the subjects to rate their ability to advance tasks during the previous week and how they felt the degree of exhaustion, e.g., “How often did the routine activities require a major effort to be completed?”, and “How often did you feel you could not carry things forward?” [85]. These tools will help healthcare providers monitor changes in intrinsic capacity over time and allow for timely interventions [86].

#### 3.2.3. Interrelation of Resilience and Intrinsic Capacity

The World Health Organization defines IC as a positive construct composed of an individual’s physical and mental capacities, which include cognition, locomotion, vitality, psychological state, and sensory function (visual and hearing) [73]. The level of functional ability an older person has at any point in time exists without regard to current acute stressors. The five-domain structure of IC has been validated in longitudinal Chinese cohort studies to serve as a strong predictive factor for the progression of basic and instrumental activities of daily living, independent of demographic variables [87]. Baseline IC scores correlate strongly with lower rates of frailty development together with improved survival results in geriatric oncology settings [88,89].

**Figure 2:**
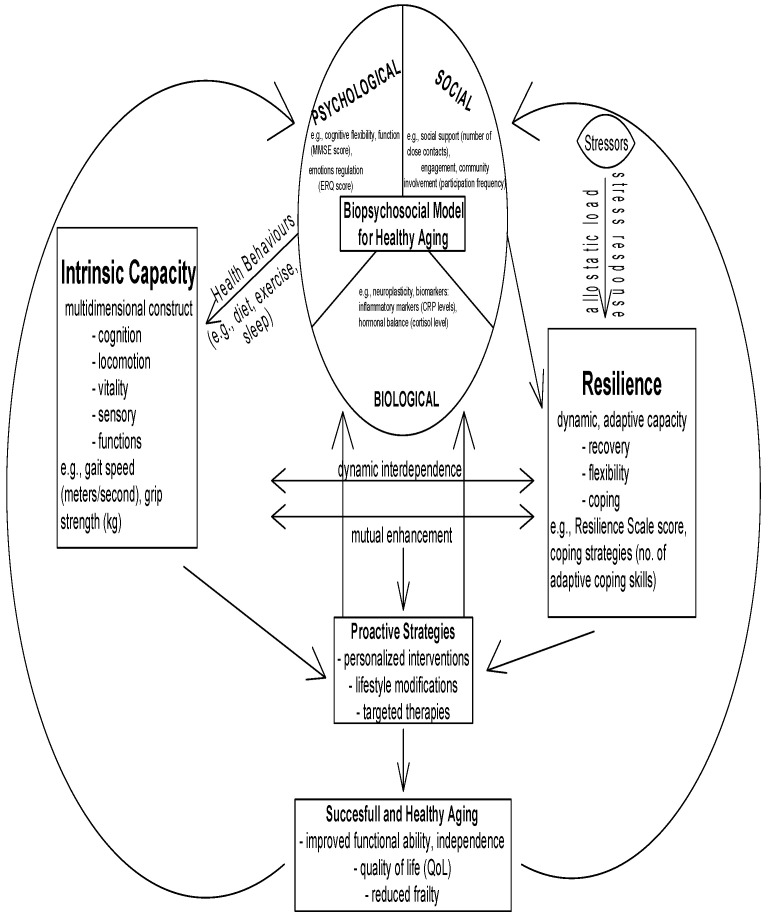
Interrelationship between resilience, intrinsic capacity and biopsychosocial framework for healthy aging.

Physical resilience represents the ability to respond to health stressors through the process of surviving or bouncing back from them at molecular, cellular, organ, or system functional levels. The fundamental difference between intrinsic capacity and resilience lies in their approach to acute stress, because resilience focuses on immediate responses while intrinsic capacity relies on baseline reserves. Resilience provides better predictive value for short-term recovery after stress exposure compared to intrinsic capacity because it specifically measures the effectiveness of reserve mobilization [90]. The interrelationship is depicted in Figure 2, illustrating the complex interactions between resilience, intrinsic capacity, and the biopsychosocial framework for healthy aging.

Better IC provides resources to handle stressors, which results in improved PR and better recovery results. People who lose their IC become unable to activate protective mechanisms against stress. Strong psychological resilience acts as a protective factor which helps maintain IC domains from stress-related deterioration and subsequently reduces the chances of cardiovascular events and functional disabilities.

The baseline composite of capacities underpins the concept of IC while resilience enables the dynamic use of those capacities when stress occurs [19,20,21,80,86,91,92,93,94,95,96,97,98,99,100,101,102,103,104,105,106,107,108,109,110,111,112,113,114,115,116,117,118,119,120,121,122,123,124,125,126,127,128,129,130,131,132]. The two concepts exist as interdependent elements because strengthening IC through exercise and nutrition and resilience through psychosocial support and cognitive-behavioral strategies will combine to enhance healthy aging and functional ability [79]. Together, these elements form a comprehensive model for healthy aging, underscoring the importance of an integrated approach.

##### How Resilience Influences Intrinsic Capacity

Psychological resilience operates as a protective element which preserves IC, according to research findings. A cross-sectional study with subjects aged 65 and above revealed that adults with high psychological resilience, according to the Sense of Coherence Scale (SOC), demonstrated a 42.9% reduced risk of developing atrial fibrillation, after controlling for potential confounders. The association demonstrates how psychological resilience maintains wide-ranging IC by protecting against atrial fibrillation’s impacts on cognitive, psychological, and physical domains [91].

Research on post-acute COVID-19 syndrome patients showed that when resilience was combined with frailty phenotypes, these factors produced significant predictions about both IC and quality-of-life scores. The odds ratio for impaired intrinsic capacity (IC) was the highest among patients who were frail yet non-resilient (OR  ≈ 7.39), and fit yet non-resilient patients had higher odds (OR ≈ 4.34) than resilient phenotypes. Research shows that psychological resilience protects IC from deterioration when people face significant physiological challenges [92].

##### How Intrinsic Capacity Supports Resilience

In a study that utilized structural equation modeling to examine hospitalized patients aged 76 on average (mean age), higher IC scores in cognitive and locomotive domains serve as predictors of better PR, which helps individuals bounce back to their previous functional state after encountering stressors. The research found that physiologic reserve had positive associations with cognitive and locomotor IC domains, which were strongly linked to PR measured by PRIFOR (Perceived Resilience Scale). The study used PRIFOR scores to forecast better outcomes for discharge functional status by evaluating frailty scale and QoL (quality of life) [93].

The geroscience framework shows how cellular and physiologic resilience decline through aging mechanisms to cause stepwise reductions in IC, leading to frailty and chronic disease. The foundation of resilience operations relies on IC to function [94].

##### Illustrative Examples

The KORA-Age study investigated a community cohort consisting of 940 participants aged between 65 and 93 years and demonstrated that disability development was inversely related to both resilience and intrinsic capacity scores over three and seven years. The overall “robustness” (non-frailty) showed the strongest predictive ability, but resilience and IC separately identified different segments of the population who were at risk for disability [90].

A China-based frailty prevention study analyzed a sample of 4000 older people who showed that better initial IC levels led to reduced frailty development over two and four years (OR~0.64). The strongest protective effects against frailty development emerged from the vitality and locomotor capacity domains, with vitality OR~0.33 and vitality + locomotor combined OR~0.11. The study demonstrates that individuals with strong intrinsic capacity demonstrate reduced vulnerability to failing under stressors because they retain resilience and reserve capabilities [88].

The social frailty cohort research showed that social isolation together with poor psychosocial functioning resulted in negative impacts on all IC domains which include cognitive and psychological function and vitality. Social challenges diminish both resilience and IC as they lead to diminished cognitive and psychological and vitality domain abilities [95].

#### 3.2.4. Intervention to Improve IC and Resilience and Future Directions

Interventions pursuing resilience and IC are essential to counteract pathological aging and to prevent functional decline. Multi-domain community-based programs promoting nutrition, exercise, and polypharmacy exhibited confidence in preventing frailty escalation and building resilience [86,96]. Recent meta-analysis confirms that decline in composite IC, particularly in locomotion, vitality, and cognition, is associated with a 11% higher mortality risk, highlighting the need for prompt interventions [97]. A 2024 meta-analysis of multicomponent nutritional and exercise interventions demonstrated a significant decrease in physical frailty. These findings validate the assurance of such programs in reducing frailty escalation and increasing adaptive capacity in older adults [98]. Tailored interventions addressing the locomotion and vitality IC domains constantly surpass classical single-target approaches.

A quasi-experimental study using texture-modified plant-based dietary supplements with Taiwanese retirees showed improvements in endurance and gait speed after four months [99,100].

A pre-post study that combined exercise with cognitive stimulation over six months in pre-frail individuals showed a relevant composite IC score increase and a reduced frailty status compared to exercise-only or control groups, suggesting the cumulated benefit of dual-domain strategies [101]. Emerging intervention methods hold great potential as shown in a multi-center study conducted in Ireland, which implemented a 3-month home-based strength exercise plus high-protein guidance (1.2 g/kg/day) among 168 older adults and achieved an 77% reduction in frailty, with gains in bone mass and grip strength. Research has proven that tablet-supported exercise programs using remote coaching are successful in practice, although participants achieved only three exercise days per week over six months, though actual physical activity gains were inconsistent. Therefore, this combined method of digital-physical regimes and home-based strength plus protein protocols revealed comparable or superior efficiency to conventional community programs, supporting adaptable resilience-building approaches [102]. But gaps remain. A recent non-randomized study which included 81 participants found that multi-domain exercise and nutrition interventions did not outperform controls in reversing pre-frailty in one year, highlighting that baseline IC, rather than the intervention alone, predicts outcomes [20].

A comprehensive summary of interventions aimed at enhancing resilience and intrinsic capacity in older adults is described in Table 5 below [20,80,103,104]. It includes both established and emerging methods with documented comparative effectiveness.

Multi-domain interventions enhance intrinsic capacity. Randomized controlled trials (RCT) show that multi-domain interventions, including tailored physical exercise, nutritional counseling, cognitive stimulation, and sensory enhancement, significantly improve intrinsic capacity (IC) metrics in older adults. A 2023 meta-analysis of 25 RCTs found improvements in Mini-Mental State Examination (+MMSE), Geriatric Depression Scale-15 (GDS-15), and Short Physical Performance Battery (+SPPB), along with gains in gait speed, grip strength, and chair-stand performance [105].

Higher IC protects against frailty and functional decline. Data from a prospective cohort of 809 community-dwelling adults demonstrated that each point increase in composite IC (scored 0–10) was linked to substantially reduced risks over 12 months: frailty progression, frailty onset among robust individuals, functional decline, and falls [99].

IC reduces disability and mortality risks. A systematic review and meta-analysis of 37 longitudinal studies of pre-frail adults compared exercise (6 months), cognitive stimulation therapy (CST, 3 months), and their combination. Both exercise alone and combined exercise + CST improved composite IC and the locomotion and psychological domains after 3 months. Only the combined arm improved cognition after 3 months; after 6 and 12 months, gains were sustained or expanded across locomotion, vitality, cognition, psychological domains, and instrumental ADLs. It was found that IC inversely correlated with impairment in basic ADLs, instrumental ADLs, and mortality risk. Maintained or improved IC over time reduced IADL impairment [106].

Higher IC predicts lower frailty and functional decline. A prospective cohort found each unit increase in composite IC reduced the 12-month risks for frailty progression, incident frailty, functional decline, and falls, independent of age or comorbidities [99].

Physical exercise supports psychological resilience. A systematic review of 20 PubMed studies confirms that even brief or low-frequency physical activity enhances psychological resilience in older adults. Both cross-sectional and longitudinal findings indicate a dose–response relationship between exercise volume and resilience scores [107].

Framework for physical resilience: PR is conceptually linked to physiological reserves across multiple systems. A 2015 systematic review outlined resilience as resistance and recovery at the cellular and whole-person levels, highlighting the need for consensus in assessing and applying this construct in interventions for aging [57].

Mind/body resilience practices enhance well-being. A 2023 systematic review of RCTs reported that mind–body interventions (such as mindfulness, yoga, and stress-management training) significantly improve psychological resilience in older adults. These practices enhance coping, cognitive flexibility, self-efficacy, and stress tolerance—key resilience factors [21].

#### 3.2.5. The Importance of Assessing Resilience and IC

The World Health Organization promotes a change from disease-centered healthcare toward healthy aging by maximizing intrinsic capacity (IC) and environmental factors. The physical resilience of individuals depends on IC domains: locomotion, vitality, and cognition, which are influenced by environmental factors [108]. Current research gaps include defining how each domain of intrinsic capacity contributes to resilience and sustainability. Previous data show that all adults with high intrinsic capacity have a better quality of life and greater independence in daily living activities. Additionally, those with higher intrinsic capacity tend to live longer compared to individuals with lower intrinsic capacity. The difference in intrinsic capacity is evident when controlling for socioeconomic conditions and availability of healthcare resources [109].

Resilience involves multiple biopsychosocial factors that influence its development and functioning. Recent studies discuss the principal element of IC, with particular attention to the vitality domain and its connections to mortality risk and physical performance [110,111]. Another study indicates the prognostic value of a detailed IC assessment, connecting different IC subdomains to pathophysiological processes like stress and inflammation Several studies showed that customized interventions aimed at the vitality and locomotion IC domains proved superior to conventional methods for building robustness and identified a gap in IC-based intervention approaches which demonstrates how IC-guided treatments could transform existing geriatric healthcare practices [80]. Social support and a sense of coherence play essential roles in building resilience as they protect people against frailty development. These findings underline resilience not only as a physical but as a dynamic interplay of multiple life aspects that determine older adults’ ability to adapt and thrive.

Multi-domain community-based programs addressing nutrition, exercise, and polypharmacy exhibited assurance in preventing frailty escalation and building resilience [75].

#### 3.2.6. Impact of CGA, Resilience, and Intrinsic Capacity on Mortality, Disability, Institutionalization, Morbidity, Quality of Life, and Polypharmacy in Older Patients

Comprehensive Geriatric Assessment (CGA), when enriched with assessments of intrinsic capacity and resilience, significantly improves survival outcomes in older adults. CGA’s multidimensional structure identifies physiological vulnerabilities and enables tailored interventions. Intrinsic capacity, particularly domains like vitality and cognition, serves as a predictor of overall health reserve, while resilience captures the dynamic ability to recover after clinical stressors such as hospitalization and has been increasingly recognized as a powerful predictor of health trajectories in older adults. A recent Lancet Healthy Longevity meta-analysis of longitudinal studies demonstrated that intrinsic capacity was inversely associated with impairments in basic (BADL) and instrumental activities of daily living (IADL) (components of CGA), as well as mortality. Importantly, maintaining or improving IC over time was linked to substantially lower odds of IADL impairment [90,106].

Intrinsic capacity domains, such as locomotion and psychological well-being, act as early indicators of decline, while resilience, particularly physical resilience, reflects the ability to recover from physical stressors. CGA-guided interventions aimed at restoring function are more effective when informed by a patient’s baseline resilience. Studies reported a reduced risk of progression to frailty and disability in CGA recipients and confirm that a decline in intrinsic capacity predicts adverse outcomes including disability, care dependency, and even death [112].

Beyond the conceptual underpinning, empirical community-based evidence demonstrates that declines across IC domains independently predict adverse events such as falls and disability [113]. In a one-year longitudinal study of community-dwelling older adults, deterioration in locomotor, psychological, and sensory domains was a significant predictor of falls. Similarly, declines in locomotor, cognitive, and psychological domains independently increased the risk of incident disability [114]. These data suggest that systematically assessing and intervening upon IC domains within geriatric evaluation can help prevent falls and functional loss.

While data on functional outcomes have been mixed in broad populations, emerging studies in specific high-risk groups, such as those with hip fractures or frailty, show promising results. In orthogeriatric patients who had a CGA performed, reduced time to surgery, decreased delirium, shortened hospital stays, and improved recovery of activities of daily living following hip fracture were observed [115]. Older adults with higher baseline resilience and intrinsic capacity had better outcomes in functional rehabilitation and activities of daily living. Together these findings indicate that embedding IC assessment into geriatric evaluation adds prognostic and interventional value, supporting early identification of functional decline and opportunities to increase intrinsic reserve.

Preventing unnecessary institutionalization is a key goal of CGA. Older adults with higher resilience and preserved intrinsic capacity are more likely to maintain independence, remain in their communities, and reduce nursing home admissions. Patients with greater physical resilience, such as faster recovery from hospitalization or surgery, had a higher likelihood of returning home and avoiding long-term care placements, validating the clinical value of resilience-informed discharge planning. Multiple randomized controlled trials and systematic reviews have demonstrated that CGA yields significant real-world benefits in enabling older adults to remain in their own homes. A landmark Cochrane review reported that hospitalized older adults receiving CGA were significantly more likely to be alive and residing in the community at 3 to 12 months follow-up, representing a clinically meaningful improvement in autonomy and independence. The same review found that CGA also significantly reduced institutionalization risk, underscoring the intervention’s capacity to preserve functional independence while potentially alleviating pressure on long-term care services [116].

Reduced physical resilience increases the risk of immobility syndrome, pressure ulcers, and other complications associated with bed rest. CGA provides opportunities for early interventions, such as nutritional support, mobilizations, and fall prevention, guided by resilience-informed assessments. Community-based CGA integrating physical resilience assessments offers a proactive strategy to reduce hospital-acquired complications and leads to earlier mobilization strategies, reducing complication rates and enhancing care planning [108]. Recent interventional work highlights that CGA can go beyond functional outcomes to improve disease-specific control. A 2024 trial of nursing-led CGA intervention in hospitalized older adults effectively enhanced control of key clinical parameters such as glycemic regulation and blood pressure, as well as overall quality of life [117]. CGA enhances older adults’ resilience, not only by preserving their functional independence and quality of life but also by improving disease management and reducing institutional dependency.

Physical resilience complements intrinsic capacity in framing health outcomes in older adults. Chhetri and colleagues postulated and empirically supported that intrinsic capacity serves as an essential determinant of physical resilience via physiologic reserve. A compelling case report of a nonagenarian who suffered severe dehydration, delirium, pneumonia, and pressure injury, but ultimately achieved almost complete recovery, illustrates how baseline IC can foster remarkable resilience even in catastrophic health events [118].

The impact on treatment adherence, polypharmacy, and quality of life is also determined in previous studies. Incorporating resilience and intrinsic capacity into CGA frameworks facilitates more individualized medication management and improves adherence. For example, reduced cognitive and psychological capacity may undermine adherence, while low resilience can amplify the adverse effects of polypharmacy. Studies found out that CGA conducted by primary care physicians led to improved alignment and reduced unplanned hospitalizations. A multidisciplinary CGA approach including resilience screening helped identify patients at risk of treatment non-adherence, enhancing medication safety and promoting deprescribing when appropriate [119].

CGA is strongly associated with improved quality of life (QoL). CGA in outpatient care settings enhanced psychological support and autonomy, with resilience acting as a buffer against age-related decline, thus improving both subjective and objective QoL outcomes. Furthermore, outpatient and community-dwelling models report enhanced quality of life and medication adherence in some contexts, with improved medication-related outcomes and better adherence to modifications in CGA-supported primary care [121,122]. Though heterogeneity remains, and evidence quality varies, these findings suggest meaningful benefits in frail or highly vulnerable subgroups (Table 6).

## 4. Discussion

This narrative review distinguishes itself from prior publications by explicitly focusing on the dynamic interrelationship between resilience, particularly physical resilience, and intrinsic capacity within a comprehensive biopsychosocial framework. While the existing literature often treats resilience and intrinsic capacity as separate constructs, or emphasizes their individual roles in aging, this manuscript emphasizes their conceptual integration, highlighting how resilience acts as a process that mobilizes baseline reserves represented by intrinsic capacity. The key knowledge gap addressed here is the need for a unified understanding of how these constructs interact mechanistically and how their assessment can be operationalized to predict and improve health trajectories in older adults. By synthesizing recent empirical studies and proposing integrated measurement approaches, this review aims to bridge the gap between conceptual models and practical application, facilitating the development of targeted, early intervention to promote healthy aging.

*Policy and clinical implications*: To translate these insights into practice, more context-specific and actionable recommendations are essential. For policymakers, establishing routine community-based screening protocols for physical resilience and intrinsic capacity, using validating tools such as the Short Physical Performance Battery (SPPB), Geriatric Depression Scale (GDS), or resilience questionnaires, could enable the early identification of at-risk populations. These protocols should be integrated into existing primary care and community health services, especially in resource-limited settings with high aging population. Clinicians can then employ tailored, multi-domain interventions, such as community programs, nutritional support, and cognitive engagement activities, to strengthen resilience and baseline reserves proactively. Furthermore, culturally adapted and easily deployable screening tools should be prioritized to ensure equitable access and sustained engagement across diverse socioeconomic and ethnic groups. By embedding these protocols into routine health checks, healthcare systems can shift from reactive, disease-centered care to proactive, preventive geriatric management.

### 4.1. Summary of Main Findings

Enhancing resilience means developing the ability to adapt alongside psychological health, which leads to successful aging and quality-of-life maintenance despite age-related challenges.

The assessment of holistic health in aging individuals relies heavily on intrinsic capacity as an essential tool. Frailty denotes an increased vulnerability in older adults due to diminished physiological reserves, manifesting as unintentional weight loss, exhaustion, weakness, slow gait, and low activity levels. These characteristics are commonly assessed via the Fried Frailty Phenotype or deficit-accumulation frailty index [25]. To support this, the Comprehensive Geriatric Assessment (CGA) is a multidimensional approach to promote this process, designed to identify and manage complex health and social needs in older adults. A robust meta-analysis demonstrated that, in hospitalized older adults, CGA significantly reduces the risk of nursing home admission, falls, pressure ulcers, delirium, and community frailty [9]. Moreover, another study highlights emerging CGA models adapted for primary care, underscoring its evolving utility beyond hospital settings [123]. The brain’s ability to adapt through neuroplasticity demonstrates how resilience develops through personal experiences and intervention-based learning. The WHO’s model provides a complete framework to analyze how these constructs produce dynamic responses to environmental factors.

#### 4.1.1. Overview of Included Studies

This review encompasses 145 articles, with 43 articles meeting the inclusion criteria based on recent publication (2020–2025), study quality, and relevance to resilience and intrinsic capacity in older adults. The studies employed a variety of designs, including randomized controlled trials (RCTs), systematic reviews, cohort studies, and methodological validation studies.

#### 4.1.2. Thematic Categorization of Findings

- Assessment Methods of Resilience and Intrinsic Capacity

Multiple studies highlighted the importance of validated tools for measuring resilience and intrinsic capacity. For resilience, instruments such as the Connor–Davidson Resilience Scale (CD-RISC), Physical Resilience Scale (PRS), and resilience questionnaires tailored for specific populations (e.g., cognitive, emotional domains) were commonly used [77,78]. Intrinsic capacity was assessed using multidimensional instruments like WHO’s ICOPE screening tools, Short Physical Performance Battery (SPPB), gait speed, handgrip strength, and cognitive tests including MMSE [72,109]. These measurement approaches allow consistent quantification and longitudinal tracking of resilience and intrinsic capacity.

b. Interventions to Enhance Resilience and Intrinsic Capacity

A significant portion of the reviewed literature focused on interventional strategies:

- Multi-domain exercise programs: Several systematic reviews and meta-analyses demonstrated that structured multicomponent exercise (aerobic, resistance, balance training) significantly improves intrinsic capacity, reducing frailty, and enhancing physical and cognitive performance [14,120,121]. For example, one study reported a 1126% increase in gait speed and a decline in frailty prevalence after >12 weeks of supervised exercise.
- Nutritional interventions: Protein supplementation alone or combined with exercise showed promise in reversing sarcopenia and reducing frailty [103,104]. Notably, prolonged interventions (>24 weeks) yielded the greatest benefits in muscle strength and functional status.
- Cognitive and psychological strategies: Cognitive training and mind–body interventions such as mindfulness and yoga improved psychological resilience and mental health outcomes [21,125]. The combination with physical activity further amplified benefits in intrinsic capacity domains.
- Integrated multi-domain approaches: Combined interventions including physical activity, nutritional support, cognitive training, and sensory enhancement effectively improved intrinsic capacity domains, with some studies indicating reductions in mortality risk and functional decline [110,111].

Notably, tailored interventions addressing specific intrinsic capacity components yielded superior improvements compared to single-target strategies [124].

c. Effectiveness of Interventions Based on Study Outcomes

- Frailty and functional outcomes: Meta-analyses report that multicomponent programs reduce frailty prevalence by approximately 20–30%, improve gait speed by 0.1–0.3 m/s, and enhance activities of daily living (ADL) performance [19,124].
- Mortality and morbidity: A systematic review and meta-analyses demonstrated that high intrinsic capacity scores are associated with a 30%–50% lower risk of mortality and functional decline over follow-up periods ranging from 6 months to 2 years [80,106].
- Resilience and recovery trajectories: Studies utilizing dynamic resilience indices, such as the recovery phenotype approach, effectively predicted post-stressor outcomes like hospital readmission, prolonged rehabilitation, and institutionalization [28,76].

### 4.2. Clinical Implications

The measurement challenges of resilience and intrinsic capacity underscore the necessity for developing standardized, validated assessment tools. Recent research shows that such tools significantly improve clinical decision-making by enabling the accurate evaluation of a patient’s functional reserves and biological aging processes [126]. Precise assessment not only helps identify individuals who are at peak health and function but also guides the development of tailored intervention programs aimed at maintaining or restoring health, thus reducing adverse outcomes. Assessment effectiveness is essential for predicting clinical trajectories, including mortality, morbidity, and frailty progression. Evidence indicates that decline in intrinsic capacity correlates strongly with increased risk of mortality and adverse health events, making early detection vital for intervention planning [97]. Consistent with that, population-based studies have revealed that lower scores on composite measures of intrinsic capacity are linked to a significantly increased risk of incident cardiovascular disease and all-cause mortality, highlighting the potential of these measures as an early warning system to prompt preventive interventions. Furthermore, the findings indicate that this association is observed across different age groups and is independent of traditional cardiovascular risk factors [127]. Likewise, in acute myocardial infarction, understanding a patient’s recovery resources guides whether invasive procedures such as coronary angiography will lead to better long-term survival and functional recovery than conservative management [127]. Moreover, the evaluation of resilience helps identify vulnerable patients who may benefit most from prehabilitation or rehabilitative strategies before undergoing surgery are associated with decreased postoperative complications and shorter hospital stays, which can substantially impact healthcare resource utilization and patient outcomes [128]. In line with this, the “Vivifrail” multicomponent exercise program, implemented in community-dwelling older adults with mild cognitive impairment (MCI)/mild dementia, has demonstrated effectiveness in enhancing intrinsic capacity, especially in domains like locomotion, cognition, and vitality [106,133].

Recovery capacity is a central decision-making factor in selecting appropriate therapeutic strategies. In the management of hip fractures, patients with higher intrinsic capacity are more likely to recover independently, experience fewer complications, and achieve better functional status [126]. Similar principles apply across various clinical contexts: in oncology, the assessment of resilience and intrinsic capacity informs choices between aggressive treatments like surgery or radiotherapy versus less invasive, supportive approaches, aiming for optimal outcomes with minimal risks [14,103]. Resilience in colorectal cancer patients was linked to both the challenges and positive aspects of their illness. Providing psychological support and practical assistance related to the illness may be essential for enhancing resilience in these individuals [125].

Studies confirm that tailored, multi-domain interventions, including physical exercise, nutritional optimization, and psychosocial support, can effectively enhance resilience, leading to improved clinical outcomes. For example, multicomponent exercise interventions have demonstrated significant reductions in hospital readmission rates and improved physical function among frail older adults [19]. Protein supplementation, alone or in combination with exercise, has shown promise in reversing sarcopenia and reducing frailty-related morbidity [104]. Importantly, early identification of resilience deficits enables clinicians to implement these strategies proactively, increasing the likelihood of effective recovery and reducing long-term disability.

Other findings also highlight the importance of psychological and social factors in enhancing intrinsic capacity. Incorporating strategies such as stress management, social support, and cognitive training can strengthen resilience. These psychological interventions may work synergistically with physical and biomarker improvements. Studies show that a holistic approach, where parameters like the neutrophil-to-leucocytes ratio (NLR), free androgen index (FAI), serum albumin, dehydroepiandrosterone sulfate (DHEA-s), and vitamin D are linked to functional gains. Addressing mental well-being alongside biological markers could optimize outcomes [129]. Compared to routine care, the relative effect sizes of attention and interpretation therapy, cyclic adjustment training, cognitive intervention, expressive therapy, positive psychological interventions, and work-environment therapy showed statistically significant improvements in resilience [130].

Knowing each patient’s recovery resources allows healthcare providers not only to select strategies that optimize outcomes, such as maximizing benefits while minimizing risks, but also to determine specific intervention points to actively increase resilience. This approach facilitates a shift from reactive to proactive management, emphasizing preventative measures and early interventions, which are essential considering the progressive nature of age-related decline. By reinforcing resilience at multiple levels, clinicians can improve functional recovery after stressors, ultimately supporting healthier, more independent aging trajectories and reducing the societal burden of disability.

### 4.3. Research Implications

The constructs of resilience and intrinsic capacity offer a dynamic and holistic approach, shifting the focus from disease management to fostering adaptive responses, functional preservation, and personalized care strategies [17,18].

The assessment of resilience and IC provides a valuable framework for identifying vulnerable individuals at risk of functional decline. Tools such as the Short Physical Performance Battery (SPPB), Geriatric Depression Scale (GDS), and various functional assessments enable clinicians to monitor these constructs effectively. By incorporating such brief, validated instruments into routine geriatric assessments, clinicians can better tailor interventions, such as targeted physical activity programs, nutritional support, mental health care, and social engagement, that directly address specific vulnerabilities and promote resilience [57,77]. This proactive approach has the potential to reduce hospitalization risks, delay frailty progression, and improve overall well-being [105,124]

Recent research emphasizes that resilience and IC are not isolated constructs but interconnected, with each influencing recovery trajectories and long-term outcomes. For example, higher baseline IC has been correlated with improved resistance to stressors, such as fractures or surgeries, and with reduced mortality risk [16,88]. Conversely, low resilience often predicts poorer recovery and increased dependence [70]. Interventions aimed at improving IC, through exercise, nutrition, and psychosocial support, have shown promising results in enhancing resilience and functional status [20,75].

Furthermore, understanding the biological underpinnings of resilience, such as neuroendocrine regulation, neuroplasticity, and immune function, opens opportunities for innovative therapeutic strategies [28,32,48]. Emerging evidence suggests that modifiable factors like inflammation, hormonal responses, and psychosocial determinants can be targeted to bolster resilience and IC, ultimately supporting healthier aging trajectories [41,45,47].

However, challenges remain in standardizing measurement tools and integrating these concepts into clinical workflows universally. The heterogeneity in assessment instruments and the limited inclusion of biological markers hinder the comparability and generalizability of findings [21,76]. Future research must focus on developing validated, easy-to-use tools tailored for diverse populations and settings, and on elucidating the mechanisms linking resilience, IC, and aging-related outcomes.

The insights gathered from this review underscore that adopting an integrated, biopsychosocial framework that evaluates resilience and IC can transform current geriatric care—shifting from reactive to preventive, personalized, and strength-based strategies [9,48]. By doing so, healthcare systems can better support the complex and heterogeneous aging process, ultimately fostering resilience, enhancing function.

The significant heterogeneity within the aging population begs the question of why some older adults preserve their cognitive abilities and robust physical status despite numerous comorbidities while others do not [72].

Recent evidence underscores the essential influence of social determinants, including education, income, ethnicity, and access to healthcare, on resilience and intrinsic capacity in older adults [13,68,134]. These factors significantly shape baseline reserves and response capabilities, often contributing to disparities in aging trajectories. Marginalized populations tend to experience higher rates of fragility, disability, and mortality, illustrating the urgent need for interventions that address these social inequities [13]. Interventions rooted in resilience and intrinsic capacity frameworks have the potential to mitigate these disparities by enhancing personal assets such as physical strength, cognitive function, and social engagement. Programs that are community-based, culturally adapted, and targeted toward vulnerable groups, such as tailored physical activity, nutritional support, and psychosocial strategies, can help bridge social gaps and promote more equitable health outcomes in aging populations [131,132].

This review advances the current understanding by explicitly highlighting the interconnection of resilience and intrinsic capacity within a biopsychosocial framework. While the prior literature has explored these concepts independently, our synthesis emphasizes their dynamic relationship and their combined potential to inform personalized interventions. Importantly, it discusses how assessments of resilience and intrinsic capacity can be tailored to social contexts, guiding targeted strategies to address health disparities. This integrative approach offers actionable insights for clinicians and policymakers aiming to develop more equitable, effective, and sustainable models of healthy aging, thus representing a meaningful contribution to the evolving field of geriatric care [135].

## 5. Conclusions

The complex relationship between resilience and intrinsic capacity serves as a vital foundation for better health and well-being among older adults. Understanding these constructs improves health management effectiveness and leads to better quality of life outcomes for aging populations. Incorporating geriatric evaluation into the assessment of resilience and intrinsic capacity has the potential to enhance health outcomes to focus on more targeted interventions. Health intervention strategies and public health policies require assessments of resilience and intrinsic capacity according to the research findings highlighted in this article. Future research should concentrate on longitudinal interdisciplinary research which will expand current understanding of the relationship between resilience and intrinsic capacity across different population groups.

Understanding physical resilience is the key to enhancing recovery in older adults; addressing geriatric syndromes may not be enough for a healthy aging process, and building physical resilience may help lessen the effects of aging and prevent outcomes like frailty and disability.

The findings of this review demonstrate the importance for healthcare systems to use integrated methods which include resilience and intrinsic capacity elements. Older adults who receive proper attention to both constructs will develop stronger health and life satisfaction, which benefits their communities.

## 6. Limitations

As a narrative review, this article is subject to inherent limitations, lacking the systematic rigor of systematic reviews or meta-analyses. The non-standardized selection of studies based on relevance and author criteria may introduce biases, such as an overrepresentation of healthier, community-dwelling older adults, since those with advanced frailty, cognitive impairment, or in institutional care are underrepresented. Additionally, the wide variety of instruments used to assess resilience and intrinsic capacity complicates direct comparison across studies, limiting mechanistic understanding. The subjective, non-systematic approach may also favor studies with positive findings, and the absence of formal quality appraisal affects the robustness of conclusions. Furthermore, many included studies are observational or cross-sectional, which restricts causal inference. Future research employing systematic reviews, meta-analyses, and rigorous quality assessments is necessary to strengthen the evidence base and clinical application.

## 7. Future Directions

There is a need for longitudinal and interventional studies that examine how resilience and IC change over time and respond to targeted interventions, and that establish standardized, validated measurement tools for IC and resilience adapted for diverse populations. Research should also aim to integrate biological, behavioral, and environmental data to elucidate mechanisms underlying the interaction between biopsychosocial factors and aging outcomes.

## Figures and Tables

**Table 1:** Conceptual Mapping of Key Terms in Aging Studies.

Term	Definition	Characteristics	Interrelationship
Resilience	The dynamic ability to adapt to stressors, bounce back from adversity, and maintain or regain stability across mental, emotional, and physical domains.	Adapts to both acute and chronic stressors, influenced by intrinsic and extrinsic factors, including social support and coping mechanisms.	Relies on intrinsic capacity, supports recovery and adaptation, enhances functional ability.
Intrinsic Capacity	A holistic measure of an individual’s physical and mental capacities, comprising domains like cognition, locomotion, vitality, and psychological well-being.	Multidimensional, focuses on baseline reserves, influenced by biological, psychological, and social factors.	Supports resilience, indicative of functional reserve and overall health.
Functional Ability	The capacity to meet daily needs, make decisions, and maintain independence, reflecting the practical application of intrinsic capacities and resilience.	Practical, day-to-day application, dependent on both intrinsic capacity and resilience, sensitive to environmental support.	Enhanced by strong intrinsic capacity and resilience, reflective of successful aging.
Successful Aging	A process of maintaining physical health, mental well-being, active engagement in life, and independence through aging.	Encompasses both intrinsic capacity and resilience, characterized by reduced chronic disease burden, high function, and quality of life.	Achieved through optimized intrinsic capacity, resilience, and supportive environments, indicating healthy aging.

**Table 2:** Acute vs. Chronic Resilience—Definitions, Indicators, and Representative Tools.

Type of Resilience	Definition	Typical Examples	Determined by	Diagnostic Scales/Tools	Purpose of the Scales
Acute Resilience	The ability to respond quickly and effectively to a sudden, singular, or intense stressor	Recovery after surgery, a fall, or a stroke	Speed of recovery, baseline functional status, nervous and hormonal system reactivity	- CFS - SPPB - Post-Acute Care Evaluation Tools	- Assess immediate functional capacity - Monitor recovery trajectory - Predict short-term outcomes
Chronic Resilience	The ability to maintain functionality and cope with a persistent or recurring stressor over time	Living with a chronic illness, long-term disability, ongoing psychosocial stress	Long-term coping mechanisms, social support, psychological factors, and lifestyle	- RS-14 or RS-25 - CD-RISC -WHOQOL-BREF	- Evaluate psychological and behavioral adaptation—Measure long-term well-being - Guide chronic care planning

Terms: CFS—Clinical frailty Scale; SPPB—Short Physical Performance Battery; RS—Resilience Scale; CD-RISC—Connor Davidson Resilience Scale; WHOQOL BREF—World Health organization Quality of Life Brief.

**Table 3:** Resilience by Stressor Type—Domains, definitions, example, and Tools.

Type of Resilience	Definition	Typical Examples	Determined by	Diagnostic Scales/Tools	Purpose of Scales
Psychological Resilience	The capacity to maintain or regain stable mental health and adapt well in the face of adversity, sustaining functioning without long-term psychological disruption	Coping with trauma, major life changes, interpersonal crises	Cognitive appraisal, self-efficacy, optimism, social support, coping skills	CD-RISC, RS-14/RS-25, Brief Resilience Scale	Assess resilience as maintenance/adaptation; evaluate interventions; predict long-term mental health outcomes
Emotional Resilience	The ability to recognize, manage, and recover from emotional stressors and regulate emotional responses effectively	Managing sadness, anger, fear, grief; sustaining equilibrium in emotional upheaval	Emotional awareness, regulation, optimism, internal/external resource use	ERQ, emotional intelligence inventories	Evaluate emotional coping, regulation capacity, and recovery from emotional disruption
General Health Resilience	A broad construct referring to the ability to maintain or return to overall good health status across domains (mental, physical, functional) in response to health challenges	Sustaining quality of life during chronic illness, multimorbidity, or after hospitalization	Multi-domain functioning: self-rated health, behavior, social support, lifestyle factors	WHOQOL-BREF, health-related quality-of-life scales, multi-domain resilience questionnaires	Assess long-term well-being, guide care planning, and evaluate adaptation across health domains
Physical Resilience	The ability to resist functional decline or recover functional abilities following physical stressors; rooted in physiological reserve and recovery trajectory	Recovering mobility after surgery or fall, restoring strength after illness	Physiological reserve, baseline function, neuromuscular and hormonal reactivity	Short Physical Performance Battery (SPPB), Clinical Frailty Scale (CFS), grip strength test, gait speed, Physical Resilience Scale	Evaluate functional capacity, Predict recovery trajectory, Guide rehabilitation planning and care decisions.

Terms: CFS—Clinical frailty Scale; SPPB—Short Physical Performance Battery; PRS—Physical Resilience Scale; CD-RISC—Connor Davidson Resilience Scale; RS-25—Resilience Scale 25 Items; RS-14—Short version of RS; ERQ—Emotion Regulation Questionnaire; WHOQOL BREF—World Health organization Quality of Life Brief.

**Table 4:** Methods for Assessing Intrinsic Capacity and Physical Resilience—Tools, Advantages, Limitations and Scopes.

Domain/Method	Assessment Tool	Advantages	Limitations	Scope/Applicable Population
Composite Intrinsic Capacity (IC)	Summed/averaged standardized domain scores	Captures multidimensional reserve; flexible across studies	Heterogeneous scoring; poor cross-study comparability; no standard reference	Research in health in older adults
Locomotion	Short Physical Performance Battery (SPPB; chair stand, gait speed, balance	Strong predictor of disability and mortality, widely validated; objective and reliable	Requires space, in-person testing, trained staff; time-consuming in high-throughput settings	Community-dwelling and clinical geriatric populations
	Gait speed test (4–6 m walk)	Validated predictor of adverse outcomes; simple, quick, robust across settings	May not fully capture mobility nuances (e.g., dynamic stability)	Used in both outpatient and inpatient older adults
Vitality	Handgrip strength (dynamometer)	Correlates with muscle mass and mortality, easy to administer	Single measure of vitality only; affected by participant effort	Broader adult and older adult cohorts
	Mini-Nutritional Assessment—Short Form (MNA—SF)	Multidimensional; validated nutritional risk screening	Less objective; may miss early declines, relies on self-report	Community-living and clinical older adults
	Appendicular skeletal muscle mass (Bioelectrical impedance Analysis)	Linked to frailty; objective quantitative measure	Affected by hydration status; equipment dependent	Research and clinical settings with equipment
Cognition	Mini-Mental State Examination (MMSE)	Widely used and normed	Ceiling/floor effects; educational/cultural bias	Primary care and specialist geriatric assessments
Psychological	Geriatric Depression Scale (GDS 10/15)	Short and feasible; validated for elderly depressive symptoms	Not resilience-specific; self-report subject to bias; mood-sensitive	Depressive screening in older adults
Sensory	Self-reported vision and hearing questionnaires	Suitable for large samples; easy, low-cost	Poor sensitivity to subclinical losses; subjective; recall bias	Population surveys and primary care
	Snellen/Tumbling E chart, audiometry	Flexible across studies; captures multidimensional reserve	No standard reference; poor cross-study comparability; heterogeneous scoring	Ophthalmology/audiology clinics
Physical Resilience (PR)	Physical Resilience Scale (PRS): 15-item self-report scale developed to assess older adults’ physical resilience in response to health stressors	Correlates significantly with general resilience measures and physical activity; demonstrated good internal consistency and moderate test–retest reliability; I Self-report	Includes few highly challenging items, making it less sensitive in individuals with high baseline resilience	Community-dwelling older adults in US housing sites (age ~76), with single-group repeated measures
	Physical resilience instrument for older adults (PRIFOR-16): 16-item multidimensional scale assessing positive thinking, ADL coping, and hope. Validated in post-acute hospital populations in Taiwan	Reflects a multidimensional conceptualization of resilience; suitable for older adults recovering from acute illness; excellent internal consistency; strong criterion validity with frailty, cognition, depression	Certain items show ceiling effects in high-functioning individuals; mostly validated in hospital-based samples in Asia; no published data on test–retest reliability or responsiveness	Vulnerable and frail ≥65-year-olds in Taiwanese hospital wards; verified for predictive validity over 1 month
	PRIFOR-4 (Short Form)	Brief; maintains conceptual focus of long-form; easy to administer in busy clinical environments	Psychometric data on test–retest reliability still limited; convergent validity modest; shortened length may reduce sensitivity to nuanced coping strategies	Designed for quick clinical screening; same hospitalized older adult group as PRIFOR-16
	Connor–Davidson Resilience Scale (CD-RISC)	Detects adaptive coping; psychometrically robust	Structure inconsistencies; self-report; cultural biases; may not reflect actual physiological resilience	General population, military, clinical groups (e.g., PTSD, surgery)
	CHEES Scale (Comprehensive High-functioning Engagement and Evaluation Scale)	Assesses engagement and resilience behaviors	Still under validation; multidimensional	Suitable for community-dwelling and clinical populations
	Residual Methods (e.g., residual distance to predicted decline)	Detects subtle changes over time	Complex modeling; requires longitudinal data	Research settings, longitudinal studies
	Dynamic Balance Recovery Measures: Limits of Stability Tests, Postural Sway Analysis	Measures balance recovery, dynamic stability	Requires specialized equipment; in-lab only	Clinical and research settings; fall-risk assessment

Terms: SPPB—Short Physical Performance Battery; MNA-SF—Mini Nutritional Assessment—Short Form; MMSE—Mini-Mental State Examination; GDS—Geriatric Depression Scale; PRS—Physical Resilience Scale; PRIFOR-16—Physical resilience instrument for older adults -16; ADL—Activities of Daily Living; PRIFOR-4—Physical resilience instrument for older adults-4; CD-RISC—Connor–Davidson Resilience Scale; CHEES Scale—Comprehensive High-functioning Engagement and Evaluation Scale; PTSD—Post-traumatic stress disorder.

**Table 5:** Summary of Interventions Targeting Enhanced Intrinsic Capacity and Resilience in Older Adults.

Intervention Type	IC Domains Targeted	Components	Key Findings	Author (Year)
Multicomponent Exercise Program	Locomotion, strength, balance, cognition	≥12 wk. supervised/group exercise combining aerobic, resistance, balance training	Significant frailty reduction; improvements in muscle strength, gait speed, balance; improved SPPB (Short Physical Performance Battery), and TUG (Timed Up and Go) score	Yang (2024) [124]
Multicomponent Exercise in Cognitive Frailty	Cognitive, locomotion, strength	Group-based aerobic + resistance >120 min/week	Improved cognition, grip strength, lower limb strength, and frailty	Luo (2024) [19]
Home-Based Strength Training + Protein (1.2 g/kg/day)	Vitality, locomotion	Resistance exercises + dietary protein guidance for 3 mo.	Frailty decreased from 17.7% to 6.3% vs. control (OR 0.23); improved grip strength and bone mass	Travers (2023) [20]
Protein Supplementation + Exercise (Meta-analysis)	Muscle mass, strength, performance	Whey/EAAs + resistance training ≥24 wk.	Improved skeletal muscle index, and improved handgrip vs. exercise only	Yoshimura (2025) [103]
Protein Alone (Meta-analysis)	Muscle mass, strength, function	Oral protein Supplementation (≥15 g/d)	No significant benefit on LBM, strength, SPPB, or gait	Oktaviana (2020) [104]

Terms: SPPB—Short Physical Performance Battery, TUG—Timed Up and Go, EAAs—Essential Amino Acids, LBM—Lean Body Mass.

**Table 6:** Summary of Key Findings on Resilience and Intrinsic Capacity in Older Adults.

Study/Source	Study Design	Population	Interventions	Targeted IC Domains	Main Outcomes	Reported Effectiveness	Duration	Frequency and Intensity	Adherence Rate
Yang et al. (2024) [124]	Systematic review and meta-analysis	Frail older adults	≥12-week multicomponent exercise	Locomotion, strength, balance, cognition	Frailty reduction, gait speed increase, muscle strength improvement	>20% reduction in frailty; significant performance gains	≥12 weeks	3 sessions/week, 60 min/session	85%
Yoshimura et al. (2025) [103]	Systematic review and meta-analysis	Older adults with sarcopenia	Protein supplementation ± exercise	Vitality, locomotion	Muscle mass and strength ↑, fall risk ↓	Notable improvements in physical function	Variable	3–4 sessions/week, moderate intensity	80%
Wu et al. (2023) [21]	Systematic review and meta-analysis	Community-dwelling older adults	Mind-body interventions (mindfulness, yoga)	Psychological, resilience	Resilience and mental health ↑	Significant psychological resilience enhancements	Variable	1–2 sessions/week, 30–60 min/session	Varies
Li et al. (2024) [97]	Meta-analysis	Community-dwelling older adults	Multi-domain interventions (exercise, nutrition, cognitive)	Multiple IC domains	Frailty, functional decline, mortality ↓	Significant improvements; lower adverse events	Variable	Varies, multi-domain interventions	75%
Luo et al. (2024) [19]	Systematic review and meta-analysis	Older adults with cognitive frailty	≥12-week multicomponent exercise	Locomotion, cognition	Gait, strength, frailty ↓	Improved physical and cognitive functions	≥12 weeks	3 sessions/week, 60 min/session	75–80%
Travers et al. (2023) [20]	RCT	Older adults	6-month multicomponent program	Physical, cognitive, nutritional	Frailty and disability ↓	77% reduction in frailty; improved quality of life	6 months	4 sessions/week, 45 min/session	80%

Terms: IC—intrinsic capacity; RCT—randomized controlled trials; ↑—increased; ↓—reduced.
